# Birth weight in relation to health and disease in later life: an umbrella review of systematic reviews and meta-analyses

**DOI:** 10.1186/s12916-016-0692-5

**Published:** 2016-09-28

**Authors:** Lazaros Belbasis, Makrina D. Savvidou, Chidimma Kanu, Evangelos Evangelou, Ioanna Tzoulaki

**Affiliations:** 1 Department of Hygiene and Epidemiology, University of Ioannina Medical School, Ioannina, Greece; 2 Academic Department of Obstetrics and Gynecology, Chelsea and Westminster Hospital, Imperial College London, London, UK; 3 Department of Epidemiology and Biostatistics, School of Public Health, Imperial College London, London, UK; 4 MRC-PHE Centre for Environment and Health, School of Public Health, Imperial College London, London, UK

**Keywords:** Bias, Birth weight, Epidemiological credibility, Meta-analysis

## Abstract

**Background:**

Birth weight, a marker of the intrauterine environment, has been extensively studied in epidemiological research in relation to subsequent health and disease. Although numerous meta-analyses have been published examining the association between birth weight and subsequent health-related outcomes, the epidemiological credibility of these associations has not been thoroughly assessed. The objective of this study is to map the diverse health outcomes associated with birth weight and evaluate the credibility and presence of biases in the reported associations.

**Methods:**

An umbrella review was performed to identify systematic reviews and meta-analyses of observational studies investigating the association between birth weight and subsequent health outcomes and traits. For each association, we estimated the summary effect size by random-effects and fixed-effects models, the 95 % confidence interval, and the 95 % prediction interval. We also assessed the between-study heterogeneity, evidence for small-study effects and excess significance bias. We further applied standardized methodological criteria to evaluate the epidemiological credibility of the statistically significant associations.

**Results:**

Thirty-nine articles including 78 associations between birth weight and diverse outcomes met the eligibility criteria. A wide range of health outcomes has been studied, ranging from anthropometry and metabolic diseases, cardiovascular diseases and cardiovascular risk factors, various cancers, respiratory diseases and allergies, musculoskeletal traits and perinatal outcomes. Forty-seven of 78 associations presented a nominally significant summary effect and 21 associations remained statistically significant at *P* < 1 × 10^−6^. Thirty associations presented large or very large between-study heterogeneity. Evidence for small-study effects and excess significance bias was present in 13 and 16 associations, respectively. One association with low birth weight (increased risk for all-cause mortality), two dose-response associations with birth weight (higher bone mineral concentration in hip and lower risk for mortality from cardiovascular diseases per 1 kg increase in birth weight) and one association with small-for-gestational age infants with normal birth weight (increased risk for childhood stunting) presented convincing evidence. Eleven additional associations had highly suggestive evidence.

**Conclusions:**

The range of outcomes convincingly associated with birth weight might be narrower than originally described under the “fetal origin hypothesis” of disease. There is weak evidence that birth weight constitutes an effective public health intervention marker.

**Electronic supplementary material:**

The online version of this article (doi:10.1186/s12916-016-0692-5) contains supplementary material, which is available to authorized users.

## Background

In early 1990’s, the “fetal origin hypothesis” of adult diseases was suggested to describe the observed associations between low birth weight (BW) and cardiovascular diseases in adult life [1–5]. Barker, who first observed these associations, hypothesized that fetal under-nutrition may lead to disproportionate fetal growth and program later coronary heart disease risk [6].

Since then, the importance of the early life and intrauterine environment in relation to later disease has been widely acknowledged and studied [1, 6–10]. BW is considered a marker of the intrauterine environment and has been extensively studied in epidemiological research, both in terms of its predictors but mainly in relation to subsequent disease. The examined phenotypes expanded beyond cardiovascular conditions into a wide range of outcomes and traits, including respiratory disease [8, 11], cancer [12, 13] and psychiatric outcomes [14]. At the same time, acknowledging its importance, WHO included low BW (<2500 g) as one of its 2025 targets, namely a 30 % reduction in the number of infants born with a BW below 2500 g by 2025 [15]. During the last two decades, interest in the potential health risks associated with high BW (>4000 g) has also emerged, and associations between high BW and the risk of adverse health outcomes have been studied in an increasing number of scientific papers.

Interpreting associations between BW and the occurrence of health problems later in life is, however, challenging and linked to a series of methodological limitations [16]. Despite the attention that BW has received in public health policy and epidemiological research, a comprehensive assessment of the proposed associations between BW and future disease is lacking. In the current study, we applied the methodology of umbrella reviews to map all the outcomes that have been associated with low and high BW and we applied a standardized approach to assess the credibility of the findings in order to identify which associations are supported by robust evidence.

## Methods

### Literature search and eligibility criteria

We performed an umbrella review, which is a comprehensive and systematic collection and evaluation of multiple systematic reviews and meta-analyses performed on a specific research topic [17]. We followed a standardized procedure that has already been applied in the appraisal of observational associations in other research fields [18–21]. We systematically searched PubMed from inception to December 24, 2015, to identify systematic reviews and meta-analyses of observational studies examining associations of BW with medical conditions, traits and biomarkers. We used the following search algorithm: (“birth weight” OR “birth size” OR “small for gestational age” OR “large for gestational age” OR “fetal growth restriction” OR “intra-uterine growth restriction”) AND (systematic review* OR systematic literature review* OR meta-analys*). We excluded meta-analyses examining genetic or environmental determinants of BW. We further excluded the meta-analyses of individual participant data that did not report the study-specific estimates and pooled analyses that only summarized evidence across a non-systematically selected number of cohort studies or that did not present the study-specific effect estimates of component studies [22–27]. We did not apply any limitation based on language of publication.

### Data extraction

Two independent researchers extracted the data (LB, CK), and in the case of discrepancies, the final decision was that of a third researcher (IT). From each eligible article, we recorded the first author, journal, year of publication, examined outcomes and number of studies included. We also extracted the study-specific effect sizes (risk ratio, odds ratio, hazard ratio, mean difference and regression coefficient) along with the corresponding 95 % confidence intervals and the number of cases and controls in each study for each association. Whenever the sample sizes were not available through the meta-analysis, we retrieved the original reports to record them. Further, when multiple comparisons were available for a particular phenotype (e.g. < 2500 g vs. ≥ 2500 g and < 2500 g vs. 2500–4000 g) we always preferred to extract information on < 2500 g versus ≥ 2500 g and > 4000 g versus ≤ 4000 g in the case of low BW and high BW, respectively. However, when this comparison was not available, we extracted the comparison reported by the meta-analysis. For the excluded meta-analyses assessing an overlapping association, we recorded the level of comparison and the summary effect estimate along with the 95 % confidence interval. Additionally, we scrutinized the full-text of the eligible papers to examine whether their authors discussed the potential effect of gestational age in the association of BW with subsequent health outcomes.

### Statistical analysis

For each meta-analysis, we estimated the summary effect size and its 95 % confidence interval with both fixed-effects and random-effects models [28, 29]. We also estimated the 95 % prediction interval, which further accounts for between-study heterogeneity and evaluates the uncertainty for the effect that would be expected in a new study addressing that same association [30, 31].

In the case of meta-analyses with continuous outcomes, the standardized mean difference was transformed to an odds ratio with an established formula [32]. Between-study heterogeneity was assessed by the I^2^ metric [33]. I^2^ ranges between 0 % and 100 % and is the ratio of between-study variance over the sum of the within-study and between-study variances [34]. Values exceeding 50 % or 75 % are usually judged to represent large or very large heterogeneity, respectively.

We assessed whether there was evidence for small-study effects (i.e. whether smaller studies tend to give substantially larger estimates of effect size compared with larger studies) with the regression asymmetry test proposed by Egger et al. [35, 36]. A *P* value less than 0.10 with a more conservative effect in the largest study than in random-effects meta-analysis was judged to be evidence for small-study effects.

We applied the excess statistical significance test, which assesses whether the observed number of studies with nominally significant results is larger than their expected number [37]. This test assesses whether the number of positive studies among those in a meta-analysis is too large based on the power that these studies have to detect plausible effects at an α of 0.05. The expected number of studies with significant results is calculated in each meta-analysis by the sum of the statistical power estimates for each component study. The power of each component study was estimated using the effect size of the largest study (smallest SE) in a meta-analysis and the power calculation was based on an algorithm using a non-central *t* distribution [38, 39]. Excess statistical significance for single meta-analyses was claimed at *P* < 0.10 [37]. For four associations, the power calculations and the excess statistical significance test were not performed, because the sample sizes of the component studies could not be retrieved neither from meta-analysis papers nor from the original reports.

Finally, we identified the associations that had the strongest validity and were not suggestive of bias. Specifically, we considered as convincing the associations that met the following criteria: significance under the random-effects model at *P* < 1 × 10^−6^, more than 1000 cases, not large between-study heterogeneity (I^2^ < 50 %), 95 % prediction interval excluding the null value, and no evidence of small-study effects and excess significance bias. Additionally, the associations with a statistically significant effect at *P* < 1 × 10^−6^, more than 1000 cases, and a statistically significant effect in the largest study were characterized as having highly suggestive evidence. We considered as suggestive the associations that have more than 1000 cases and a statistically significant effect under the random-effects model at *P* < 1 × 10^−3^. The rest of statistically significant associations at *P* < 0.05 under random-effects model were graded as weak associations.

The statistical analyses were performed with STATA version 12.0 and the power calculations were performed using STATA version 12.0 and G*Power version 3.1.

## Results

**Fig. 1:**
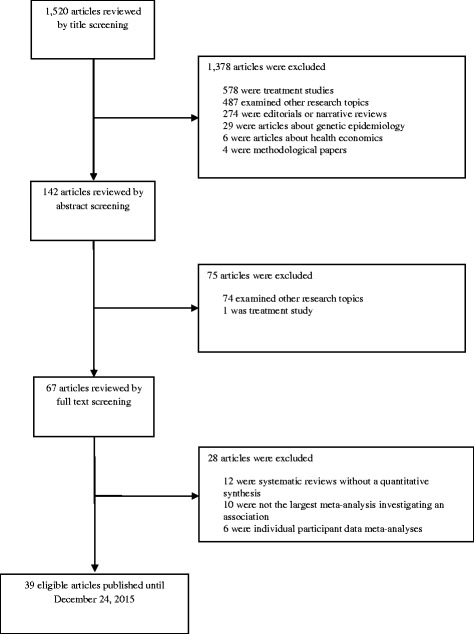
Flow chart of literature search

Overall, the literature search identified 1520 articles, of which 39 articles, published between 2005 and 2015, were deemed eligible (Fig. 1). Sixty-three papers were screened by full-text. Of these, 10 examined the same or related phenotypes in the same population (defined as overlapping meta-analysis), six were individual participant data meta-analyses that did not report the study-specific effect estimates, and 12 were systematic reviews without a quantitative synthesis. The 39 eligible papers included 78 different meta-analyses (Table 1): 28 assessing low BW, four assessing small-for-gestational age infants, 18 assessing high BW, and 28 assessing a dose–response association between BW and subsequent health outcomes. A wide range of health outcomes has been studied ranging from anthropometry and metabolic disease, cardiovascular disease and cardiovascular risk factors, various cancers, respiratory diseases and allergies, musculoskeletal traits, and perinatal outcomes. Both neonatal and childhood conditions as well as adult diseases have been extensively examined (Table 1). Only two eligible papers had access to raw data of primary studies and performed an individual-level data meta-analysis [8, 40].

**Table 1:** Quantitative synthesis, bias assessment and credibility assessment of 74 associations between different comparisons of birth weight and health outcomes or traits

Reference	Outcome	Level of comparison	N cases/ N controls	N datasets	Effect size metric	Random-effects meta-analysis (95 % CI)	*P* value (random)	I^2^	95 % prediction intervals	*P* value for Egger test	*P* value for Excess significance test
Araujo de Franca, 2014 [59]	Waist circumference	Per 1 kg increase	4898^d^	6	Regression coefficient	−0.10 (−0.73 to 0.53)	0.760	73.2	−2.04 to 1.84	0.725	0.943
Araujo de Franca, 2014 [59]	Waist-to-hip ratio	Per 1 kg increase	5008^d^	10	Regression coefficient	−0.59 (−0.84 to −0.34)	4.0 × 10^−6^	0	−0.89 to −0.30	0.545	0.052
Baird, 2011 [48]	BMC in hip	Per 1 kg increase	1795^d^	6	Regression coefficient	1.42 (0.90 to 1.94)	8.3 × 10^−8^	5.9	0.56 to 2.28	0.584	0.838
Baird, 2011 [48]	BMC in lumbar spine	Per 1 kg increase	3181^d^	7	Regression coefficient	1.72 (0.76 to 2.67)	4.2 × 10^−4^	33.5	−0.53 to 3.97	0.069^a^	1.6 × 10^−4^
Baird, 2011 [48]	BMD in hip	Per 1 kg increase	3188^d^	7	Regression coefficient	0.01 (0.00 to 0.02)	0.235	0	−0.01 to 0.01	0.708	0.271
Baird, 2011 [48]	BMD in lumbar spine	Per 1 kg increase	3506^d^	8	Regression coefficient	0.00 (−0.01 to 0.01)	0.779	14.9	−0.02 to 0.02	0.831	0.341
Berhan, 2014 [46]	Perinatal mortality in developing countries	<2500 g vs. ≥ 2500 g	21,184/285,970	14	OR	9.59 (6.11 to 15.04)	7.5 × 10^−23^	98.9	1.43 to 64.18	0.954	0.707
Caughey, 2009 [13]	Acute lymphoblastic leukaemia	<2500 g vs. ≥ 2500 g	4805/765,827	10	RR	0.97 (0.81 to 1.16)	0.736	0	0.78 to 1.20	0.289	NP
Caughey, 2009 [13]	Acute lymphoblastic leukaemia	>4000 g vs. ≤ 4000 g	11,082/2,228,906	23	RR	1.29 (1.17 to 1.42)	1.9 × 10^−7^	36.3	0.97 to 1.72	0.055^a^	9.4 × 10^−7^
Caughey, 2009 [13]	Acute lymphoblastic leukaemia	Per 1 kg increase	7404/858,650	16	RR	1.19 (1.10 to 1.28)	1.7 × 10^−5^	54.7	0.93 to 1.52	0.990	2.2 × 10^−4^
Caughey, 2009 [13]	All types of leukaemia	<2500 g vs. ≥ 2500 g	5766/766,202	11	RR	1.04 (0.86 to 1.26)	0.683	10	0.76 to 1.43	0.357	NP
Caughey, 2009 [13]	All types of leukaemia	>4000 g vs. ≤ 4000 g	NA/NA	14	RR	1.42 (1.26 to 1.60)	1.2 × 10^−8^	28.5	1.07 to 1.88	0.001^a^	NA
Caughey, 2009 [13]	All types of leukaemia	Per 1 kg increase	11,313/861,710	21	RR	1.19 (1.12 to 1.27)	5.1 × 10^−8^	45.3	0.97 to 1.45	0.590	0.001
Caughey, 2009 [13]	Acute myeloid leukaemia	<2500 g vs. ≥ 2500 g	756/851,204	9	RR	1.46 (0.87 to 2.43)	0.151	43.7	0.39 to 5.43	0.606	NP
Caughey, 2009 [13]	Acute myeloid leukaemia	>4000 g vs. ≤ 4000 g	756/851,204	9	RR	1.25 (1.09 to 1.43)	1.3 × 10^−3^	43.9	0.88 to 1.77	0.216	3.1 × 10^−4^
Chen, 2012 [60]	Bone tumour	>4000 g vs. NBW	4044/272,354	8	OR	1.21 (0.97 to 1.50)	0.091	37.8	0.71 to 2.03	0.093^a^	0.396
Christian, 2013 [40]	Childhood stunting	SGA vs. AGA (BW ≥ 2500 g)	5413/16,663	10	OR	1.92 (1.75 to 2.12)	1.1 × 10^−41^	0	1.72 to 2.15	0.968	NP
Christian, 2013 [40]	Childhood stunting	SGA vs. AGA (BW < 2500 g)	843/1171	10	OR	3.00 (2.36 to 3.81)	2.3 × 10^−19^	56.6	1.53 to 5.85	0.555	NP
Cook, 2010 [58]	Testicular cancer	<2500 g vs. NBW	6906/612,741	17	OR	1.34 (1.08 to 1.67)	7.9 × 10^−3^	50.9	0.69 to 2.62	0.135	0.049
Davey-Smith, 2007 [61]	Maternal cardiovascular mortality	Per 1 SD increase	2976/974,667	6	HR	0.75 (0.67 to 0.84)	3.1 × 10^−7^	79.8	0.53 to 1.06	0.657	0.119
Davey-Smith, 2007 [61]	Paternal cardiovascular mortality	Per 1 SD increase	9375/774,325	3	HR	0.93 (0.91 to 0.95)	2.0 × 10^−9^	0	0.80 to 1.09	0.317	0.516
der Voort, 2014 [8]	Pre-school wheezing	<2500 g vs. NBW	40,603/103,271	26	OR	1.10 (1.00 to 1.21)	0.051	16	0.89 to 1.36	0.829	NP
der Voort, 2014 [8]	School-age asthma	<2500 g vs. NBW	11,729/121,198	16	OR	1.13 (1.01 to 1.27)	0.032	0	1.00 to 1.28	0.639	NP
Dodds, 2012 [62]	Muscle strength	Per 1 kg increase	20,461^d^	14	Regression coefficient	0.86 (0.58 to 1.15)	1.9 × 10^−9^	56.4	−0.01 to 1.74	0.965	NP
Harder, 2007 [7]	Type 2 diabetes mellitus	<2500 g vs. ≥ 2500 g	5815/100,759	10	OR	1.32 (1.06 to 1.64)	0.013	60.8	0.71 to 2.43	0.196	NP
Harder, 2007 [7]	Type 2 diabetes mellitus	>4000 g vs. ≤ 4000 g	6005/108,400	9	OR	1.27 (1.01 to 1.59)	0.044	68.2	0.62 to 2.58	0.817	NP
Harder, 2008 [63]	Astrocytoma	<2500 g vs. ≥ 2500 g	1574/160,146	6	OR	0.85 (0.58 to 1.25)	0.410	31.1	0.35 to 2.09	0.931	NP
Harder, 2008 [63]	Astrocytoma	>4000 g vs. ≤ 4000 g	1812/1,649,625	8	OR	1.38 (1.07 to 1.79)	0.014	57.5	0.66 to 2.88	0.632	0.160
Harder, 2008 [63]	Medulloblastoma	<2500 g vs. ≥ 2500 g	747/158,163	5	OR	1.65 (0.42 to 6.50)	0.475	88.2	0.01 to 223	0.828^a^	0.503
Harder, 2008 [63]	Medulloblastoma	>4000 g vs. ≤ 4000 g	853/1,647,552	7	OR	1.28 (1.02 to 1.59)	0.033	5.6	0.90 to 1.80	0.818	NP
Harder, 2009 [64]	Type 1 diabetes mellitus	<2500 g vs. ≥ 2500 g	5236/1,385,809	8	OR	0.82 (0.55 to 1.24)	0.344	91.4	0.20 to 3.31	0.067	NP
Harder, 2009 [64]	Type 1 diabetes mellitus	>4000 g vs. ≤ 4000 g	6406/2,388,046	10	OR	1.17 (1.09 to 1.26)	1.2 × 10^−5^	0	1.08 to 1.28	0.883	NP
Harder, 2010 [65]	Neuroblastoma	<2500 g vs. ≥ 2500 g	2907/2,156,535	10	OR	1.24 (0.99 to 1.55)	0.058	30.2	0.74 to 2.07	0.731	NP
Harder, 2010 [65]	Neuroblastoma	>4000 g vs. ≤ 4000 g	2856/2,156,654	10	OR	1.19 (1.04 to 1.36)	0.013	0	1.01 to 1.39	0.517	NP
Jackson 2013 [66]	Pneumonia in childhood	<2500 g vs. ≥ 2500 g	1281/1464	4	OR	3.18 (1.02 to 9.91)	0.046	95.6	0.01 to 714	0.366	NP
Kormos, 2013 [67]	Intelligence in adolescence	<2500 g vs. NBW	NA/NA	15	OR^c^	0.35 (0.27 to 0.45)	1.7 × 10^−16^	75.4	0.14 to 0.84	0.001^a^	7.8 × 10^−4^
Lawlor 2005 [68]	FEV_1_	Per 1 kg increase	5438^d^	7	Regression coefficient	0.06 (0.03 to 0.08)	1.4 × 10^−5^	36.5	−0.01 to 0.12	0.242	NP
Lawlor 2006 [69]	Total cholesterol in men	Per 1 kg increase	33,650^d^	34	Regression coefficient	−0.04 (−0.07 to −0.01)	0.018	49.8	−0.15 to 0.07	0.377	NP
Lawlor 2006 [69]	Total cholesterol in women	Per 1 kg increase	23,129^d^	34	Regression coefficient	−0.01 (−0.04 to 0.02)	0.510	27.7	−0.09 to 0.07	0.686	NP
Loret de Mola, 2014 [55]	Depression in adulthood	SGA vs. AGA	397/2844	5	OR	1.14 (0.64 to 2.03)	0.656	49.3	0.20 to 6.36	0.791	NP
Mebrahtu, 2015 [11]	Wheezing disorders in childhood	<2500 g vs. ≥ 2500 g	145,421/665,431	20	OR	1.61 (1.39 to 1.85)	1.1 × 10^−10^	82.3	0.92 to 2.80	0.021^a^	NP
Mebrahtu, 2015 [11]	Wheezing disorders in childhood	>4000 g vs. NBW	44,988/736,940	10	OR	1.02 (1.00 to 1.05)	0.100	0	0.99 to 1.05	0.985	NP
Michos, 2007 [70]	Testicular cancer	>4000 g vs. NBW	5684/123,120	10	OR	1.14 (0.99 to 1.31)	0.075	42.8	0.80 to 1.62	0.791	0.006
Milne, 2013 [56]	Acute lymphoblastic leukaemia	SGA vs. AGA	6835/11,689	12	OR	1.24 (1.13 to 1.36)	4.9 × 10^−6^	0	1.12 to 1.37	0.101	NP
Mu, 2012 [71]	Diastolic blood pressure	<2500 g vs. ≥ 2500 g	29,192^d^	15	OR^c^	4.45 (1.32 to 14.99)	0.016	99.3	0.02 to 902	0.085^a^	0.001
Mu, 2012 [71]	Systolic blood pressure	<2500 g vs. ≥ 2500 g	32,351^d^	19	OR^c^	7.45 (2.19 to 25.33)	1.3 × 10^−3^	99.4	0.02 to 2296	0.148	0.615
Mu, 2014 [41]^b^	Asthma in adulthood	<2500 g vs. ≥ 2500 g	2111/37,409	4	OR	1.25 (1.12 to 1.40)	7.7 × 10^−5^	0	0.98 to 1.60	0.884	NP
Øglund, 2015 [72]	Physical activity	Per 1 kg increase	10,667^d^	8	Regression coefficient	−3.08 (−10.20 to 4.04)	0.397	9	−14.81 to 8.66	0.881	NP
Papadopoulou, 2012 [73]	Hodgkin lymphoma in childhood	<2500 g vs. NBW	669/64,058	3	OR	0.94 (0.54 to 1.65)	0.829	0	0.03 to 35.25	0.344	NP
Papadopoulou, 2012 [73]	Non-Hodgkin lymphoma in childhood	<2500 g vs. NBW	1571/68,265	5	OR	1.07 (0.71 to 1.63)	0.740	55.4	0.29 to 3.95	0.599	NP
Papadopoulou, 2012 [73]	Non-Hodgkin lymphoma in childhood	>4000 g vs. NBW	1615/297,469	6	OR	1.17 (0.76 to 1.81)	0.473	66.7	0.32 to 4.28	0.085^a^	0.207
Panduru, 2013 [74]	Atopic dermatitis	<2500 g vs. NBW	6315/100,663	10	OR	0.66 (0.48 to 0.90)	9.1 × 10^−3^	85.6	0.23 to 1.87	0.558	0.412
Panduru, 2013 [74]	Atopic dermatitis	>4000 g vs. NBW	6224/62,672	6	OR	1.13 (0.97 to 1.31)	0.108	62.9	0.74 to 1.73	0.426	NP
Risnes, 2011 [42]^b^	All-cause mortality	<3000 g vs. NBW	32,926/276,648	8	HR	1.12 (1.07 to 1.16)	6.9 × 10^−8^	21.7	1.03 to 1.21	0.642	NP
Risnes, 2011 [42]^b^	All-cause mortality	>4000 g vs. NBW	32,926/276,648	8	HR	1.02 (0.98 to 1.05)	0.302	0	0.98 to 1.06	0.317	NP
Risnes, 2011 [42]^b^	All-cause mortality	Per 1 kg increase	36,834/361,874	18	HR	0.94 (0.92 to 0.97)	2.6 × 10^−6^	18.6	0.89 to 0.99	0.838	0.038
Risnes, 2011 [42]^b^	Mortality from cardiovascular diseases	Per 1 kg increase	11,366/314,715	16	HR	0.88 (0.85 to 0.91)	1.6 × 10^−13^	0	0.84 to 0.91	0.717	0.177
Risnes, 2011 [42]^b^	Mortality from cancer	Per 1 kg increase	9233/269,944	10	HR	1.09 (1.04 to 1.14)	3.4 × 10^−4^	20.8	0.99 to 1.19	0.276	0.037
Schellong, 2012 [43]^b^	Overweight/obese in adulthood	<2500 g vs. ≥ 2500 g	73,420/330,648	30	OR	0.67 (0.59 to 0.76)	1.1 × 10^−9^	82	0.38 to 1.18	0.079^a^	NP
Schellong, 2012 [43]^b^	Overweight/obese in adulthood	>4000 g vs. ≤ 4000 g	96,296/396,381	45	OR	1.68 (1.58 to 1.79)	3.6 × 10^−57^	74.9	1.24 to 2.28	0.133	NP
Silveira 2008 [75]	Metabolic syndrome	<2500 g vs. NBW	NA/NA	11	OR	2.54 (1.57 to 4.09)	1.4 × 10^−4^	39.2	0.75 to 8.60	0.204	NA
Shi, 2015 [76]	RSV-related acute lower respiratory infection in childhood	<2500 g vs. NBW	3383/155,872	5	OR	1.91 (1.45 to 2.53)	5.9 × 10^−6^	59.1	0.81 to 4.54	0.881	NP
van Montfoort, 2005 [77]	Cortisol levels	Per 1 kg increase	2301^d^	11	Regression coefficient	−20.49 (−35.97 to −5.00)	9.5 × 10^−3^	46.3	−61.37 to 20.4	0.524	NP
Wang, 2014 [9]	Coronary heart disease	<2500 g vs. ≥ 2500 g	13,089/360,209	16	OR	1.22 (1.13 to 1.31)	4.7 × 10^−7^	7	1.08 to 1.37	0.039^a^	0.751
Wang, 2014 [9]	Coronary heart disease	>4000 g vs. ≤ 4000 g	18,243/313,235	14	OR	0.89 (0.81 to 0.98)	0.019	47.4	0.68 to 1.16	0.226	NP
Wang, 2014 [9]	Coronary heart disease	Per 1 kg increase	NA/NA	23	OR	0.82 (0.78 to 0.86)	2.4 × 10^−15^	41.1	0.70 to 0.96	0.014^a^	NA
Whincup, 2008 [78]	Type 2 diabetes mellitus	Per 1 kg increase	6090/145,994	31	OR	0.80 (0.72 to 0.88)	1.8 × 10^−5^	66.5	0.52 to 1.21	0.286	0.355
White, 2009 [79]	Chronic kidney disease	<2500 g vs. NBW	NA/NA	21	OR	1.73 (1.44 to 2.08)	8.3 × 10^−9^	66.3	0.88 to 3.38	0.015^a^	NA
Wojcik, 2013 [14]^b^	Depression in adulthood	<2500 g vs. ≥ 2500 g	9013/50,428	18	OR	1.15 (1.00 to 1.32)	0.057	34.3	0.79 to 1.67	0.171	NP
Xu, 2009 [80]	Breast cancer	Per 1 kg increase	16,299/3,604,802	16	OR	1.08 (1.03 to 1.13)	2.8 × 10^−3^	22.5	0.97 to 1.20	0.452	4.1 × 10^−4^
Yang, 2014 [12]	Colorectal cancer	Per 1 kg increase	5985/723,087	5	RR	1.05 (0.93 to 1.19)	0.461	57.1	0.72 to 1.52	0.457	0.149
Yang, 2014 [12]	Endometrial cancer	Per 1 kg increase	3780/671,410	5	RR	0.91 (0.81 to 1.03)	0.128	40.2	0.65 to 1.27	0.867	0.758
Yang, 2014 [12]	Lung cancer	Per 1 kg increase	5207/696,742	4	RR	1.09 (1.02 to 1.16)	0.016	12.3	0.90 to 1.31	0.172	0.436
Yang, 2014 [12]	Melanoma	Per 1 kg increase	4000/3,821,122	6	RR	1.14 (1.05 to 1.24)	1.9 × 10^−3^	0	1.01 to 1.29	0.912	NP
Yang, 2014 [12]	Non-Hodgkin lymphoma	Per 1 kg increase	2056/626,082	3	RR	1.12 (1.01 to 1.24)	0.033	0	0.58 to 2.15	0.487	NP
Yang, 2014 [12]	Ovarian cancer	Per 1 kg increase	2880/805,887	5	RR	0.96 (0.88 to 1.04)	0.295	0	0.83 to 1.10	0.609	NP
Zhang, 2013 [81]	Diastolic blood pressure	>4000 g vs. NBW	150,980^d^	23	MD	0.19 (−0.23 to 0.62)	0.367	72.5	−1.39 to 1.79	0.792	NP
Zhang, 2013 [81]	Systolic blood pressure	>4000 g vs. NBW	151,935^d^	24	MD	−0.25 (−0.92 to 0.42)	0.466	79.3	−2.97 to 2.47	0.477	NP

^a^Both criteria for presence of small-study effects fulfilled (*P* value for Egger’s test < 0.10 and largest study with a smaller [more conservative] effect size than random-effects summary effect size)

^b^The highlighted papers performed a comparison between studies adjusting for and not adjusting for gestational age

^c^Random-effects summary effect size estimated from standardized mean difference transformed to odds ratio

^d^In meta-analyses with a continuous outcome, the total sample size is reported

*AGA* adequate-for-gestational age, *BMC* bone mineral concentration, *BMD* bone mineral density, *CI* confidence interval, *FEV*_*1*_ forced expiratory volume in the first second, *HR* hazard ratio, *OR* odds ratio, *RR* risk ratio, *MD* mean difference, *NA* not available, *NBW* normal birth weight, *NP* not pertinent (because the number of expected significant studies was larger than the number of observed significant studies), *RSV* respiratory syncytial virus, *SD* standard error, *SGA* small-for-gestational age

Overall, we identified more than one published meta-analysis for 25 outcomes, i.e. meta-analysis examining the same exposure (birth weight) and the same outcome. Overlapping meta-analyses provided concordant results, with the exception of two pairs, which had a summary effect in opposite direction (diastolic blood pressure and overweight) compared to the meta-analysis included in our umbrella review (largest most recently published meta-analysis). Six other meta-analyses differed in the summary effect significance compared to the most recent one (Additional file 1: Table S1).

### Associations with low BW

Across 28 meta-analyses examining low BW as a dichotomous trait, the median number of cases was 5766 (interquartile range (IQR), 1574–11,729), while the median number of datasets was 11 (IQR, 8–16). Overall, 21 out of 28 associations had more than 1000 cases, 17 of 28 meta-analyses presented a nominally significant effect (*P* < 0.05) and 10 of them had a significant effect at *P* < 0.001. Only seven meta-analyses, examining the association of low BW with perinatal mortality in developing countries, wheezing disorders in childhood, being overweight or obese in adulthood, coronary heart disease, intelligence in adolescence, all-cause mortality, and chronic kidney disease, were statistically significant at *P* < 1 × 10^−6^ under the random-effects model (Table 1). The largest study had a standard error of less than 0.10 in 17 meta-analyses and a more conservative effect compared to random-effects model in 15 meta-analyses. Four meta-analyses (perinatal mortality in developing countries, coronary heart disease, school-age asthma, all-cause mortality) had a 95 % prediction interval excluding the null value. Five associations had large heterogeneity estimates (I^2^ ≥ 50 % and I^2^ ≤ 75 %), and 10 associations had very large heterogeneity estimates (I^2^ > 75 %). On bias assessment, seven associations had evidence for small-study effects (chronic kidney disease, coronary heart disease, diastolic blood pressure, intelligence in adolescence, medulloblastoma, wheezing disorders in childhood, and being overweight or obese in adulthood), and four associations (chronic kidney disease, diastolic blood pressure, intelligence in adolescence, and testicular cancer) had hints for excess significance bias (Table 1, Additional file 2: Table S2).

### Associations with high BW

Across 18 meta-analyses examining high BW as a dichotomous trait, the median number of cases was 6115 (IQR, 3153–10,642), 16 meta-analyses were supported by more than 1000 cases, and the median number of datasets was 10 (IQR, 8–14). Ten associations presented a significant effect at *P* < 0.05, but only three associations (acute lymphoblastic leukaemia, all types of leukaemia, and being overweight or obese in adulthood) remained statistically significant after the application of a more conservative significance threshold (*P* < 1 × 10^−6^). The largest study had a standard error of less than 0.10 in four meta-analyses and a more conservative effect compared to random-effects model in 12 meta-analyses. Only four meta-analyses (all types of leukaemia, neuroblastoma, type 1 diabetes mellitus, and being overweight or obese in adulthood) had a 95 % prediction interval excluding the null value (Table 1). The heterogeneity estimate was large (I^2^ ≥ 50 % and I^2^ ≤ 75 %) in seven meta-analyses and only one meta-analysis presented very large heterogeneity (I^2^ > 75 %). Two associations presented hints for both small-study effects and excess significance bias (acute lymphoblastic leukaemia and all types of leukaemia), another two associations had only small-study effects (bone tumour and non-Hodgkin lymphoma in childhood), and two additional associations had hints for excess significance bias (acute myeloid leukaemia and testicular cancer).

### Dose–response associations with BW

Across 28 meta-analyses, the median number of cases was 6747 (IQR, 3945–11,326) and the median number of datasets was 8 (IQR, 6–16). Overall, 17 associations were significant at *P* < 0.05, but only six associations survived in the application of a more stringent *P* value (*P* < 1 × 10^−6^). The largest study had a standard error of less than 0.10 in 21 meta-analyses and a more conservative effect compared to the random-effects model in 20 meta-analyses. Only six associations (all-cause mortality, bone mineral concentration in hip, coronary heart disease, melanoma, mortality from cardiovascular diseases, and waist-to-hip ratio) presented 95 % prediction interval excluding the null value (Table 1). Five associations presented large heterogeneity, and one association had very large heterogeneity. Hints for small-study effects and excess statistical significance were present in two (bone mineral concentration in lumbar spine, coronary heart disease) and eight meta-analyses (all-cause mortality, acute lymphoblastic leukaemia, all types of leukaemia, bone mineral concentration in lumbar spine, breast cancer, coronary heart disease, mortality from cancer, and waist-to-hip ratio), respectively (Table 1, Additional file 2: Table S2).

### BW relative to gestational age

Three papers performed four meta-analyses examining associations between small-for-gestational-age infants (defined as BW below the 10th percentile for the gestational age) and the risk for acute lymphoblastic leukaemia, childhood stunting and depression. No meta-analyses on large-for-gestational age infants were identified. Under the random-effects model, three associations had a statistically significant effect at *P* < 1 × 10^−6^ and 95 % prediction interval excluding the null value (acute lymphoblastic leukaemia and childhood stunting in infants with low and normal BW; Table 1). Only one association had large between-study heterogeneity, whereas none of the examined associations presented evidence for small-study effects or excess significance bias.

Despite the importance of gestational age on BW, only four out of the 36 papers (pertained to seven meta-analyses) examining low BW, high BW or dose–response relationships with BW, presented subgroup analyses, including only studies that provided gestational age-adjusted estimates (Table 1) [14, 41–43]. None of these analyses observed a statistically significant difference in the summary effect between the studies adjusting for gestational age and the unadjusted studies. Additionally, 18 (46 %) papers mentioned that the observed effect might differ from the true effect because gestational age was not considered as an adjustment variable in several observational studies. Twenty papers (51 %) reported the observational studies that adjusted for gestational age in the statistical models.

### Assessment of epidemiological credibility

Twenty-eight of 78 associations (36 %) did not present a significant summary effect at *P* < 0.05. Of the remaining 50 associations, only four presented convincing evidence by having more than 1000 cases, not large heterogeneity, 95 % prediction interval excluding the null value, a significant summary effect at *P* < 1 × 10^−6^, and absence of small-study effects and excess significance bias (Table 2). These associations pertained to all-cause mortality for low versus normal BW, bone mineral concentration in hip and mortality from cardiovascular diseases per 1 kg increase in BW, and childhood stunting for small- versus adequate-for-gestational-age infants with BW ≥ 2500 g. Notably, apart from the meta-analyses on stunting, which included gestational age in the definition of the examined phenotype (small-for-gestational-age), none of the other three meta-analyses with convincing evidence restricted their analyses to studies with adjustment for gestational age. Eleven additional associations had highly suggestive evidence (more than 1000 cases, a significant summary effect at *P* < 1 × 10^−6^ and largest study with a significant effect). These associations examined perinatal mortality in developing countries, wheezing disorders, being overweight or obese in adulthood, coronary heart disease for the comparison of < 2500 g versus ≥2500 g, intelligence in adolescence for the comparison of low BW versus normal BW, all types of leukaemia, being overweight or obese in adulthood for the comparison of > 4000 g versus ≤ 4000 g, muscle strength and coronary heart disease for the comparison of increase per 1 kg in BW, and maternal cardiovascular mortality and paternal cardiovascular mortality for the comparison of increase per 1 SD in BW. Fourteen associations presented suggestive evidence and 13 associations had weak evidence (Table 2).

**Table 2:** Summary of evidence grading for meta-analyses associating different contrasts of birth weight and risk of future disease

Level of evidence	Criteria	Low birth weight vs. normal birth weight		High birth weight vs. normal birth weight		Per unit/SD increase		SGA vs. AGA
		Increased risk in LBW group	Decreased risk in LBW group	Increased risk in HBW group	Decreased risk in HBW group	Increased risk per unit/SD increase	Decreased risk per unit/SD increase	Increased risk in SGA group
Convincing	>1000 cases, *P* < 1 × 10^−6^, I^2^ < 50 %, 95 % PI excluding the null value, no small-study effects and excess significance bias	All-cause mortality	None	None	None	BMC in hip	Mortality from cardiovascular diseases	Childhood stunting (in BW ≥ 2500 g group)
Highly suggestive	>1000 cases, *P* < 1 × 10^−6^, largest study with a statistically significant effect	Perinatal mortality in developing countries, wheezing disorders in childhood, coronary heart disease	Overweight or obese in adulthood, intelligence in adolescence	All types of leukaemia, overweight or obese in adulthood	None	None	Muscle strength, coronary heart disease, maternal cardiovascular mortality, paternal cardiovascular mortality	None
Suggestive	>1000 cases, *P* < 1 × 10^−3^	Asthma in adulthood, RSV-related acute lower respiratory infections in childhood, chronic kidney disease	None	Acute lymphoblastic leukaemia, type 1 diabetes mellitus	None	BMC in lumbar spine, acute lymphoblastic leukaemia, all types of leukaemia, FEV_1_, mortality from cancer	Waist-to-hip ratio, all-cause mortality, type 2 diabetes mellitus	Acute lymphoblastic leukaemia
Weak	The remaining associations with *P* < 0.05	Diastolic blood pressure, testicular cancer, type 2 diabetes mellitus, metabolic syndrome, pneumonia in childhood, school-age asthma, systolic blood pressure	Atopic dermatitis	Acute myeloid leukaemia, astrocytoma, medulloblastoma, neuroblastoma, type 2 diabetes mellitus	Coronary heart disease	Breast cancer at any age, lung cancer, melanoma, non-Hodgkin lymphoma	Cortisol levels, total cholesterol in men	Childhood stunting (in BW < 2500 g group)

*AGA* adequate-for-gestational age, *BMC* bone mineral concentration, *BW* birth weight, *FEV*_*1*_ forced expiratory volume in the first second, *HBW* high birth weight, *LBW* low birth weight, *SD* standard deviation, *SGA* small-for-gestational age, *RSV* respiratory syncytial virus

## Discussion

Our work constitutes the first comprehensive mapping and appraisal of the association between BW and the risk of subsequent health outcomes, as provided by published systematic reviews and meta-analyses of observational studies. Overall, 78 associations have been examined, including a diverse range of outcomes: cardiovascular, cancer, metabolic, respiratory and mortality outcomes, and disease traits and biomarkers. Despite common belief that the intrauterine environment as assessed by BW is associated with many diseases and disease traits in adult life [1, 6–10], our comprehensive assessment shows that convincing evidence only exists between the associations of low BW and increased risk for all-cause mortality, per 1 kg increase in BW and higher bone mineral concentration in hip and lower risk for mortality from cardiovascular diseases. Furthermore, the association between small-for-gestational-age and childhood stunting in low- and middle-income countries was supported by convincing evidence. There was no convincing evidence supporting associations between high BW and later outcomes; however, the associations with overweight or obesity in later life and all types of leukaemia were highly suggestive.

The associations between BW and cardiovascular disease were amongst the first to be observed in the medical literature [1–5] and our data suggests that the current evidence is highly suggestive. Both meta-analyses looking at low (<2500 g) versus high (≥4000 g) BW and those examining per 1 SD increase in BW showed highly significant summary effects and small between-study heterogeneity. However, both associations presented evidence for small-study effects and the dose–response association additionally had hints for excess significance bias. The latter may have resulted in inflated effect estimates for an association with cardiovascular disease that needs cautious interpretation [35, 44]. Despite the fact that studies have adjusted for a range of confounders, including socioeconomic status, not all studies were adjusted for gestational age, which is an important confounder and this, as well as other unrecognized confounders, could explain the observed association. In addition, the mechanisms underlying this association remain unclear despite many hypotheses having been suggested, including the one supporting that intrauterine under-nutrition leads to fetal adaptation, which is subsequently related to adverse cardiovascular risk in later life [10]. However, others have provided evidence that at least some of the association between the BW of individuals and their later risk of cardiovascular disease may be genetic and therefore not modifiable via interventions that target the intrauterine environment [45]. The causal pathway linking BW to cardiovascular risk needs further elucidation to allow evidence-based public health interventions.

The observed increased risk of cardiovascular disease associated with lower BW is likely to be a main contributor to the inverse association of BW with all-cause mortality; an association supported by convincing evidence in our assessment [42]. The higher incidence of perinatal mortality in the low BW group is also likely contributing to the all-cause mortality association with low BW, but only to a small extent. Babies born with a BW below 2500 g had increased perinatal mortality, an association supported by a very large summary effect estimate and a very small *P* value [46]. However, the meta-analysis on perinatal mortality was focused exclusively on developing countries. Therefore, the effect estimate might be exaggerated due to lack of neonatal intensive care units or difficult access to specialized healthcare facilities in these countries [47]. These data could not be generalised to other settings where high-quality healthcare is available.

The association between low BW and low bone mineral concentration in later life is less well studied compared to other outcomes and current data stem from six studies contributing to the meta-analysis [48]. Despite the fact that the association with bone mineral concentration in hip showed convincing evidence, cautious interpretation is required as data on osteoporotic fractures has not been reviewed and meta-analyses on other anatomical sites (e.g. lumbar spine) showed evidence for excess significance bias and no convincing associations.

Comparisons between BW and later overweight and obesity do not support a detrimental health effect of low BW. BW less than 2500 g was found to be protective for being overweight or obese, whereas BW greater than 4000 g was linked with an increased risk for being overweight or obese in adult life [43]. These associations were supported by highly suggestive evidence, but they also displayed very large between-study heterogeneity. Heterogeneity could be due to biased results in some of the included studies, but it could also reflect genuine differences across studies [35]. BW distributions are remarkably different across developed and developing countries [49], and the associations between BW and later adiposity may differ in these populations, contributing to the heterogeneity of the observed results. High BW is potentially causally associated with maternal BMI and glucose levels [50, 51]; however, the extent to which it could be modified through lifestyle or pharmacological interventions merits further investigation, particularly through long-term follow-up of interventions during pregnancy, which will strengthen and enhance the available evidence, particularly between high BW and subsequent risk of childhood and adulthood obesity [52–54].

Although 29 associations focused on outcomes related to different types of cancer, high BW was found to be a risk factor only for developing leukaemia [13]. The associated summary effect estimate might be inflated by the presence of small-study effects and excess significance bias. However, the statistical heterogeneity was not large, the 95 % prediction interval excluded the null value and the association was highly significant. Similarly, despite diabetes being central in the “fetal origin hypothesis” [7], its association with high and low BW has weak evidence in the literature and is only suggestive of a direct association with high BW in line with the obesity-associated evidence.

Despite intensive research on BW reflected by the large number of meta-analyses identified, there were only three papers that performed meta-analyses of studies assessing low BW in relation to gestational age [40, 55, 56], whereas no single meta-analysis on large-for-gestational-age neonates was identified. As BW and gestational age are highly correlated, analyses which consider size-for-gestational-age rather than BW adjusted for gestational age have been proposed as a more appropriate alternative [57, 58]. Among the examined phenotypes in relation to small-for-gestational-age, the association between small-for-gestational-age without low BW and childhood stunting in low- and middle-income countries showed convincing evidence. However, those results require cautious interpretation as the analyses were stratified by BW and the association between small-for-gestational age with low BW and childhood stunting showed a much weaker effect estimate and was only supported by weak evidence. Additionally, those analyses focused on low- and middle-income countries, limiting the generalisability of those results but at the same time also highlighting the need for interventions during the pregnancy period in these populations [40]. The remaining meta-analyses included a mixture of studies that adjusted or not their analyses for gestational age and, hence, the current literature is inconclusive on the effects of BW relative to gestational age.

In the present study, we applied the umbrella review approach summarising data from already published systematic reviews and meta-analyses. This approach takes full advantage of the existing meta-analyses to perform a standardised methodological process for the assessment of the epidemiological credibility of the findings. However, our study has some caveats. First, the Egger test and excess statistical significance test offer hints of bias, and not proof thereof, while the Egger test is difficult to interpret when the between-study heterogeneity is large. Further, our excess significance estimates were based on the largest study of each meta-analysis and they might be conservative, because often these studies were not necessarily very large or might have had inherent biases themselves. Furthermore, we did not appraise the quality of the primary studies, because this was beyond the scope of this umbrella review. This should be the aim of the original systematic reviews and meta-analyses, which should examine the methodological characteristics of the component studies.

## Conclusions

Our study maps the current status of evidence on 78 associations of BW with various health outcomes, traits and biomarkers. Of them, only three examined the effects of BW in relation to gestational age through size-at-birth defined phenotypes. Our results show that the range of outcomes associated with BW is narrow and smaller than described under the fetal origin of disease hypothesis. Currently, there is weak evidence that BW constitutes an effective public policy intervention for long-term health and disease.

## Additional files

Additional file 1: Table S1. Overlapping associations examined in older papers and/or under different levels of comparison. BMC: bone mineral concentration, BMD: bone mineral density, BMI: body mass index, MD: mean difference, NBW: normal birth weight, OR: odds ratio, RR: risk ratio [82–90]. (DOC 229 kb) Additional file 2: Table S2. Comparison of random-effects summary effect size and largest study effect size, expected and observed number of significant associations and excess significance test in each meta-analysis. †Random-effects summary effect size estimated from standardized mean difference transformed to odds ratio. AGA: adequate-for-gestational age, BMC: bone mineral concentration, BMD: bone mineral density, CI: confidence interval, FEV: forced expiratory volume in the first second, HR: hazard ratio, OR: odds ratio, RR: risk ratio, MD: mean difference, RSV: respiratory syncytial virus, SD: standard error, SE: standard error, SGA: small-for-gestational age. (DOC 447 kb)

## Abbreviations

BW: Birth weight
IQR: Inter-quartile range
