# Supplementary material for: Birth weight in relation to health and disease in later life: an umbrella review of systematic reviews and meta-analyses
Source: BMC Med. 2016 Sep 28;14:147. doi: 10.1186/s12916-016-0692-5 (PMC5039803; doi:10.1186/s12916-016-0692-5)
Supplement: Additional file 1: Table S1. — Overlapping associations examined in older papers and/or under different levels of comparison. BMC: bone mineral concentration, BMD: bone mineral density, BMI: body mass index, MD: mean difference, NBW: normal birth weight, OR: odds ratio, RR: risk ratio [82–90]. (DOC 229 kb) [file 12916_2016_692_MOESM1_ESM.doc]

**Additional file 1: Table S1.** Overlapping associations examined in older papers and/or under different levels of comparison

| **Outcome** | **Reference of eligible meta-analysis / Reference of older meta-analysis** | **Level of comparison** | **Effect size metric** | **Summary effect size (95% CI) in older meta-analysis** | **Concordant direction** | **Significant effect** | |
| --- | --- | --- | --- | --- | --- | --- | --- |
|  |  |  |  |  |  | **In this analysis** | **In older meta-analysis** |
| Acute lymphoblastic leukemia | [13]/[82] | >4,000g vs. ≤4,000g | OR | 1.22 (1.10 to 1.35) | Yes | Yes | Yes |
| All types of leukemia | [13]/[82] | >4,000g vs. ≤4,000g | OR | 1.34 (1.17 to 1.53) | Yes | Yes | Yes |
| Acute myeloid leukemia | [13]/[82] | >4,000g vs. ≤4,000g | OR | 1.27 (0.73 to 2.20) | Yes | Yes | No |
| Asthma in childhood | [8]/[83] | <2,500g vs. ≥2,500g | RR | 1.16 (1.13 to 1.20) | Yes | Yes | Yes |
| Asthma in childhood | [8]/[41] | <2,500g vs. ≥2,500g | OR | 1.28 (1.09 to 1.50) | Yes | Yes | Yes |
| Asthma in childhood | [11]/[84] | >4,000g vs. ≤4,000g | RR | 1.20 (1.10 to 1.30) | Yes | Yes | Yes |
| BMC in lumbar spine | [48]/[85] | Per 1 kg increase | Regression coefficient | 0.86 (-0.39 to 2.11) | Yes | Yes | No |
| BMD in lumbar spine | [48]/[85] | Per 1 kg increase | Regression coefficient | 0.004 (-0.01 to 0.01) | Yes | No | No |
| BMI | [43]/[86] | <2,500g vs. ≥2,500g | MD | -0.42 (-0.63 to -0.21) | Yes | Yes | Yes |
| BMI | [43]/[86] | >4,000g vs. ≤4,000g | MD | 0.79 (0.61 to 0.97) | Yes | Yes | Yes |
| Coronary heart disease | [9]/[87] | Per 1 kg increase | RR | 0.84 (0.81 to 0.88) | Yes | Yes | Yes |
| Depression in adulthood | [14]/[55] | <2,500g vs. ≥2,500g | OR | 1.39 (1.21 to 1.60) | Yes | No | Yes |
| Diastolic blood pressure | [81]/[71] | >4,000g vs. ≤4,000g | MD | -0.37 (-1.19 to 0.45) | No | No | No |
| Hypertension | [71]/[71] | <2,500g vs. ≥2,500g | OR | 1.21 (1.13 to 1.30) | Yes | Yes | Yes |
| Hypertension | [81]/[71] | >4,000g vs. ≤4,000g | OR | 0.78 (0.71 to 0.86) | Yes | No | Yes |
| Obesity | [43]/[88] | >4,000g vs. ≤4,000g | OR | 2.07 (1.91 to 2.24) | Yes | Yes | Yes |
| Obesity | [43]/[88] | <2,500g vs. ≥2,500g | OR | 0.61 (0.46 to 0.80) | Yes | Yes | Yes |
| Overweight | [43]/[86] | <2,500g vs. ≥2,500g | OR | 1.12 (0.90 to 1.40) | No | Yes | No |
| Overweight | [43]/[86] | >4,000g vs. ≤4,000g | OR | 1.43 (1.25 to 1.64) | Yes | Yes | Yes |
| Total cholesterol | [69]/[89] | Per 1 kg increase | Regression coefficient | -1.39 (-1.81 to -0.97) | Yes | Yes | Yes |
| Total cholesterol | [69]/[90] | Per 1 kg increase | Regression coefficient | -0.05 (-0.08 to -0.02) | Yes | Yes | Yes |
| Type 2 diabetes mellitus | [7]/ [7] | >4,000g vs. NBW | OR | 1.36 (1.07 to 1.73) | Yes | Yes | Yes |
| Type 2 diabetes mellitus | [7]/ [7] | <2,500g vs. NBW | OR | 1.47 (1.26 to 1.72) | Yes | Yes | Yes |
| Systolic blood pressure | [81]/[71] | >4,000g vs. ≤4,000g | MD | -2.08 (-2.98 to -1.17) | Yes | No | Yes |
| Wheezing disorders in childhood | [11]/[11] | <2,500g vs. NBW | OR | 1.37 (1.05 to 1.79) | Yes | Yes | Yes |

BMC: bone mineral concentration, BMD: bone mineral density, BMI: body mass index, CI: confidence interval, MD: mean difference, NBW: normal birth weight, OR: odds ratio, RR: risk ratio
