# Supplementary material for: Birth weight in relation to health and disease in later life: an umbrella review of systematic reviews and meta-analyses
Source: BMC Med. 2016 Sep 28;14:147. doi: 10.1186/s12916-016-0692-5 (PMC5039803; doi:10.1186/s12916-016-0692-5)
Supplement: Additional file 2: Table S2. — Comparison of random-effects summary effect size and largest study effect size, expected and observed number of significant associations and excess significance test in each meta-analysis. †Random-effects summary effect size estimated from standardized mean difference transformed to odds ratio. AGA: adequate-for-gestational age, BMC: bone mineral concentration, BMD: bone mineral density, CI: confidence interval, FEV: forced expiratory volume in the first second, HR: hazard ratio, OR: odds ratio, RR: risk ratio, MD: mean difference, RSV: respiratory syncytial virus, SD: standard error, SE: standard error, SGA: small-for-gestational age. (DOC 447 kb) [file 12916_2016_692_MOESM2_ESM.doc]

**Additional file 2: Table S2.** Comparison of random-effects summary effect size and largest study effect size, expected and observed number of significant associations and excess significance test in each meta-analysis.

| **Reference** | **Outcome** | **Level of comparison** | **Effect size metric** | **Random-effects summary effect size (95% CI)** | **P-value (random)** | **Largest study effect size (95% CI)** | **SE** | **Observed significant studies** | **Expected significant studies** | **P-value for excess significance test** |
| --- | --- | --- | --- | --- | --- | --- | --- | --- | --- | --- |
| Araujo de Franca, 2014[59] | Waist circumference | Per 1 kg increase | Regression coefficient | -0.10 (-0.73 to 0.53) | 0.760 | 0.64 (0.16 to 1.12) | 0.245 | 2 | 1.92 | 0.943 |
| Araujo de Franca, 2014[59] | Waist-to-hip ratio | Per 1 kg increase | Regression coefficient | -0.59 (-0.84 to -0.34) | 4.0 × 10^-6^ | -0.37 (-0.83 to 0.09) | 0.235 | 3 | 1.09 | 0.052 |
| Baird, 2011[48] | BMC in hip | Per 1 kg increase | Regression coefficient | 1.42 (0.90 to 1.94) | 8.3 × 10^-8^ | 0.96 (0.04 to 1.88) | 0.469 | 4 | 3.76 | 0.838 |
| Baird, 2011[48] | BMC in lumbar spine | Per 1 kg increase | Regression coefficient | 1.72 (0.76 to 2.67) | 4.2 × 10^-4^ | 0.42 (-0.75 to 1.59) | 0.597 | 4 | 0.81 | 1.6 × 10^-4^ |
| Baird, 2011[48] | BMD in hip | Per 1 kg increase | Regression coefficient | 0.01 (0.00 to 0.02) | 0.235 | 0.00 (-0.01 to 0.01) | 0.006 | 1 | 0.36 | 0.271 |
| Baird, 2011[48] | BMD in lumbar spine | Per 1 kg increase | Regression coefficient | 0.00 (-0.01 to 0.01) | 0.779 | 0.00 (-0.02 to 0.01) | 0.008 | 1 | 0.41 | 0.341 |
| Berhan, 2014[46] | Perinatal mortality in developing countries | <2,500g vs. ≥2,500g | OR | 9.59 (6.11 to 15.04) | 7.5 × 10^-23^ | 14.36 (13.15 to 15.68) | 0.045 | 14 | 13.86 | 0.707 |
| Caughey, 2009[13] | Acute lymphoblastic leukemia | <2,500g vs. ≥2,500g | RR | 0.97 (0.81 to 1.16) | 0.736 | 0.73 (0.50 to 1.06) | 0.192 | 0 | 7.26 | NP |
| Caughey, 2009[13] | Acute lymphoblastic leukemia | >4,000g vs. ≤4,000g | RR | 1.29 (1.17 to 1.42) | 1.9 × 10^-7^ | 1.05 (0.90 to 1.23) | 0.080 | 8 | 1.76 | 9.4 × 10^-7^ |
| Caughey, 2009[13] | Acute lymphoblastic leukemia | Per 1 kg increase | RR | 1.19 (1.10 to 1.28) | 1.7 × 10^-5^ | 1.09 (0.99 to 1.21) | 0.051 | 7 | 2.05 | 2.2 × 10^-4^ |
| Caughey, 2009[13] | All types of leukemia | <2,500g vs. ≥2,500g | RR | 1.04 (0.86 to 1.26) | 0.683 | 0.73 (0.50 to 1.06) | 0.192 | 0 | 8.02 | NP |
| Caughey, 2009[13] | All types of leukemia | >4,000g vs. ≤4,000g | RR | 1.42 (1.26 to 1.60) | 1.2 × 10^-8^ | 1.20 (1.01 to 1.42) | 0.087 | 8 | NA | NA |
| Caughey, 2009[13] | All types of leukemia | Per 1 kg increase | RR | 1.19 (1.12 to 1.27) | 5.1 × 10^-8^ | 1.09 (0.99 to 1.21) | 0.051 | 8 | 2.85 | 1.1 × 10^-3^ |
| Caughey, 2009[13] | Acute myeloid leukemia | <2,500g vs. ≥2,500g | RR | 1.46 (0.87 to 2.43) | 0.151 | 2.20 (1.10 to 4.40) | 0.354 | 2 | 7.68 | NP |
| Caughey, 2009[13] | Acute myeloid leukemia | >4,000g vs. ≤4,000g | RR | 1.25 (1.09 to 1.43) | 1.3 × 10^-3^ | 1.05 (0.90 το 1.23) | 0.080 | 3 | 0.51 | 3.1 × 10^-4^ |
| Chen, 2012[60] | Bone tumor | >4,000g vs. NBW | OR | 1.21 (0.97 to 1.50) | 0.091 | 1.02 (0.79 to 1.31) | 0.129 | 1 | 0.45 | 0.396 |
| Christian, 2013 [40] | Childhood stunting | SGA vs. AGA (BW≥2,500g) | OR | 1.92 (1.75 to 2.12) | 1.1 × 10^-41^ | 2.06 (1.77 to 2.41) | 0.079 | 9 | 9.57 | NP |
| Christian, 2013 [40] | Childhood stunting | SGA vs. AGA (BW<2,500g) | OR | 3.00 (2.36 to 3.81) | 2.3 × 10^-19^ | 3.83 (3.12 to 4.70) | 0.105 | 7 | 7.71 | NP |
| Cook, 2010[58] | Testicular cancer | <2,500g vs. NBW | OR | 1.34 (1.08 to 1.67) | 7.9 × 10^-3^ | 1.07 (0.84 to 1.38) | 0.127 | 4 | 1.62 | 0.049 |
| Davey-Smith, 2007[61] | Maternal cardiovascular mortality | Per 1 SD increase | HR | 0.75 (0.67 to 0.84) | 3.1 × 10^-7^ | 0.71 (0.67 to 0.75) | 0.029 | 5 | 3.09 | 0.119 |
| Davey-Smith, 2007[61] | Paternal cardiovascular mortality | Per 1 SD increase | HR | 0.93 (0.91 to 0.95) | 2.0 × 10^-9^ | 0.93 (0.90 to 0.95) | 0.014 | 2 | 1.44 | 0.516 |
| der Voort, 2014[8] | Pre-school wheezing | <2,500g vs. NBW | OR | 1.10 (1.00 to 1.21) | 0.051 | 1.16 (1.03 to 1.30) | 0.059 | 4 | 10.67 | NP |
| der Voort, 2014[8] | School-age asthma | <2,500g vs. NBW | OR | 1.13 (1.01 to 1.27) | 0.032 | 1.12 (0.95 to 1.32) | 0.084 | 0 | 3.84 | NP |
| Dodds, 2012[62] | Muscle strength | Per 1 kg increase | Regression coefficient | 0.86 (0.58 to 1.15) | 1.9 × 10^-9^ | 0.42 (0.04 to 0.80) | 0.194 | 8 | 10.72 | NP |
| Harder, 2007[7] | Type 2 diabetes mellitus | <2,500g vs. ≥2,500g | OR | 1.32 (1.06 to 1.64) | 0.013 | 1.64 (1.46 to 1.84) | 0.059 | 3 | 8.12 | NP |
| Harder, 2007[7] | Type 2 diabetes mellitus | >4,000g vs. ≤4,000g | OR | 1.27 (1.01 to 1.59) | 0.044 | 1.24 (0.96 to 1.61) | 0.132 | 4 | 5.28 | NP |
| Harder, 2008[63] | Astrocytoma | <2,500g vs. ≥2,500g | OR | 0.85 (0.58 to 1.25) | 0.410 | 0.81 (0.53 to 1.23) | 0.215 | 0 | 2.21 | NP |
| Harder, 2008[63] | Astrocytoma | >4,000g vs. ≤4,000g | OR | 1.38 (1.07 to 1.79) | 0.014 | 1.26 (0.96 to 1.65) | 0.138 | 5 | 3.07 | 0.160 |
| Harder, 2008[63] | Medulloblastoma | <2,500g vs. ≥2,500g | OR | 1.65 (0.42 to 6.50) | 0.475 | 1.12 (0.66 to 1.92) | 0.272 | 1 | 0.54 | 0.503 |
| Harder, 2008[63] | Medulloblastoma | >4,000g vs. ≤4,000g | OR | 1.28 (1.02 to 1.59) | 0.033 | 1.43 (0.99 to 2.08) | 0.189 | 0 | 3.32 | NP |
| Harder, 2009[64] | Type 1 diabetes mellitus | <2,500g vs. ≥2,500g | OR | 0.82 (0.55 to 1.24) | 0.344 | 0.44 (0.40 to 0.49) | 0.052 | 2 | 8 | NP |
| Harder, 2009[64] | Type 1 diabetes mellitus | >4,000g vs. ≤4,000g | OR | 1.17 (1.09 to 1.26) | 1.2 × 10^-5^ | 1.17 (1.05 to 1.31) | 0.056 | 2 | 4.56 | NP |
| Harder, 2010[65] | Neuroblastoma | <2,500g vs. ≥2,500g | OR | 1.24 (0.99 to 1.55) | 0.058 | 1.24 (0.90 to 1.69) | 0.161 | 1 | 3.95 | NP |
| Harder, 2010[65] | Neuroblastoma | >4,000g vs. ≤4,000g | OR | 1.19 (1.04 to 1.36) | 0.013 | 1.26 (0.92 to 1.72) | 0.160 | 0 | 4.36 | NP |
| Jackson 2013[66] | Pneumonia in childhood | <2,500g vs. ≥2,500g | OR | 3.18 (1.02 to 9.91) | 0.046 | 10.30 (7.80 to 13.60) | 0.142 | 3 | 3.96 | NP |
| Kormos, 2013[67] | Intelligence in adolescence | <2,500g vs. NBW | OR† | 0.35 (0.27 to 0.45) | 1.7 × 10^-16^ | 0.61 (0.53 to 0.70) | 0.068 | 13 | 6.54 | 7.8 × 10^-4^ |
| Lawlor 2005[68] | FEV_1_ | Per 1 kg increase | Regression coefficient | 0.06 (0.03 to 0.08) | 1.4 × 10^-5^ | 0.03 (0.00 to 0.06) | 0.014 | 5 | 6.93 | NP |
| Lawlor 2006[69] | Total cholesterol in men | Per 1 kg increase | Regression coefficient | -0.04 (-0.07 to -0.01) | 0.018 | -0.09 (-0.11 to -0.06) | 0.012 | 4 | 6.64 | NP |
| Lawlor 2006[69] | Total cholesterol in women | Per 1 kg increase | Regression coefficient | -0.01 (-0.04 to 0.02) | 0.510 | -0.01 (-0.04 to 0.03) | 0.018 | 3 | 7.32 | NP |
| Loret de Mola, 2014 [55] | Depression in adulthood | SGA vs. AGA | OR | 1.14 (0.64 to 2.03) | 0.656 | 0.92 (0.51 to 1.66) | 0.301 | 0 | 0.32 | NP |
| Mebrahtu, 2015[11] | Wheezing disorders in childhood | <2,500g vs. ≥2,500g | OR | 1.61 (1.39 to 1.85) | 1.1 × 10^-10^ | 1.23 (1.17 to 1.28) | 0.023 | 9 | 9.16 | NP |
| Mebrahtu, 2015[11] | Wheezing disorders in childhood | HBW vs. NBW | OR | 1.02 (1.00 to 1.05) | 0.100 | 1.02 (0.99 to 1.04) | 0.013 | 0 | 1.07 | NP |
| Michos, 2007[70] | Testicular cancer | >4,000g vs. NBW | OR | 1.14 (0.99 to 1.31) | 0.075 | 0.96 (0.82 to 1.12) | 0.080 | 3 | 0.74 | 6.4 × 10^-3^ |
| Milne, 2013 [56] | Acute lymphoblastic leukemia | SGA vs. AGA | OR | 1.24 (1.13 to 1.36) | 4.9 × 10^-6^ | 1.38 (1.13 to 1.67) | 0.099 | 3 | 9.17 | NP |
| Mu, 2012[71] | Diastolic blood pressure | <2,500g vs. ≥2,500g | OR† | 4.45 (1.32 to 14.99) | 0.016 | 1.20 (0.99 to 1.46) | 0.098 | 7 | 2.41 | 1.3 × 10^-3^ |
| Mu, 2012[71] | Systolic blood pressure | <2,500g vs. ≥2,500g | OR† | 7.45 (2.19 to 25.33) | 1.3 × 10^-3^ | 1.50 (1.24 to 1.82) | 0.098 | 9 | 7.94 | 0.615 |
| Mu, 2014[41] | Asthma in adulthood | <2,500g vs. ≥2,500g | OR | 1.25 (1.12 to 1.40) | 7.7 × 10^-5^ | 1.25 (1.11 to 1.40) | 0.059 | 1 | 2.51 | NP |
| Øglund, 2015[72] | Physical activity | Per 1 kg increase | Regression coefficient | -3.08 (-10.20 to 4.04) | 0.397 | -0.76 (-12.04 to 10.52) | 5.755 | 1 | 4.51 | NP |
| Papadopoulou, 2012[73] | Hodgkin lymphoma in childhood | <2,500g vs. NBW | OR | 0.94 (0.54 to 1.65) | 0.829 | 1.23 (0.58 to 2.60) | 0.383 | 0 | 1.01 | NP |
| Papadopoulou, 2012[73] | Non-Hodgkin lymphoma in childhood | <2,500g vs. NBW | OR | 1.07 (0.71 to 1.63) | 0.740 | 1.25 (0.80 to 1.95) | 0.227 | 1 | 2.28 | NP |
| Papadopoulou, 2012[73] | Non-Hodgkin lymphoma in childhood | >4,000g vs. NBW | OR | 1.17 (0.76 to 1.81) | 0.473 | 0.90 (0.66 to 1.22) | 0.157 | 2 | 0.90 | 0.207 |
| Panduru, 2013[74] | Atopic dermatitis | <2,500g vs. NBW | OR | 0.66 (0.48 to 0.90) | 9.1 × 10^-3^ | 0.86 (0.77 to 0.97) | 0.059 | 5 | 3.74 | 0.412 |
| Panduru, 2013[74] | Atopic dermatitis | >4,000g vs. NBW | OR | 1.13 (0.97 to 1.31) | 0.108 | 1.17 (1.06 to 1.29) | 0.050 | 3 | 3.05 | NP |
| Risnes, 2011[42] | All-cause mortality | <3,000g vs. NBW | HR | 1.12 (1.07 to 1.16) | 6.9 × 10^-8^ | 1.13 (1.08 to 1.17) | 0.020 | 3 | 4.73 | NP |
| Risnes, 2011[42] | All-cause mortality | >4,000g vs. NBW | HR | 1.02 (0.98 to 1.05) | 0.302 | 1.03 (0.98 to 1.09) | 0.027 | 0 | 1.28 | NP |
| Risnes, 2011[42] | All-cause mortality | Per 1 kg increase | HR | 0.94 (0.92 to 0.97) | 2.6 × 10^-6^ | 0.95 (0.92 to 0.99) | 0.019 | 7 | 3.53 | 0.038 |
| Risnes, 2011[42] | Mortality from cardiovascular diseases | Per 1 kg increase | HR | 0.88 (0.85 to 0.91) | 1.6 × 10^-13^ | 0.90 (0.84 to 0.96) | 0.034 | 7 | 4.56 | 0.177 |
| Risnes, 2011[42] | Mortality from cancer | Per 1 kg increase | HR | 1.09 (1.04 to 1.14) | 3.4 × 10^-4^ | 1.06 (0.99 to 1.14) | 0.036 | 4 | 1.59 | 0.037 |
| Schellong, 2012[43] | Overweight/obese in adulthood | <2,500g vs. ≥2,500g | OR | 0.67 (0.59 to 0.76) | 1.1 × 10^-9^ | 0.72 (0.68 to 0.78) | 0.035 | 18 | 23.83 | NP |
| Schellong, 2012[43] | Overweight/obese in adulthood | >4,000g vs. ≤4,000g | OR | 1.68 (1.58 to 1.79) | 3.6 × 10^-57^ | 1.56 (1.50 to 1.63) | 0.021 | 32 | 33.25 | NP |
| Silveira 2008[75] | Metabolic syndrome | <2,500g vs. NBW | OR | 2.54 (1.57 to 4.09) | 1.4 × 10^-4^ | 1.80 (1.00 to 3.50) | 0.320 | 5 | 5.42 | NP |
| Shi, 2015[76] | RSV-related acute lower respiratory infection in childhood | <2,500g vs. NBW | OR | 1.91 (1.45 to 2.53) | 5.9 × 10^-6^ | 1.70 (1.50 to 2.00) | 0.073 | 4 | 4.65 | NP |
| van Montfoort, 2005[77] | Cortisol levels | Per 1 kg increase | Regression coefficient | -20.49 (-35.97 to -5.00) | 9.5 × 10^-3^ | -21.90 (-38.20 to -5.50) | 8.342 | 5 | 7.13 | NP |
| Wang, 2014[9] | Coronary heart disease | <2,500g vs. ≥2,500g | OR | 1.22 (1.13 to 1.31) | 4.7 × 10^-7^ | 1.12 (1.01 to 1.24) | 0.052 | 5 | 4.43 | 0.751 |
| Wang, 2014[9] | Coronary heart disease | >4,000g vs. ≤4,000g | OR | 0.89 (0.81 to 0.98) | 0.019 | 0.94 (0.87 to 1.02) | 0.041 | 2 | 2.72 | NP |
| Wang, 2014[9] | Coronary heart disease | Per 1kg increase | OR | 0.82 (0.78 to 0.86) | 2.4 × 10^-15^ | 0.92 (0.86 to 1.00) | 0.038 | 11 | NA | NA |
| Whincup, 2008[78] | Type 2 diabetes mellitus | Per 1kg increase | OR | 0.80 (0.72 to 0.88) | 1.8 × 10^-5^ | 0.82 (0.77 to 0.89) | 0.037 | 10 | 7.77 | 0.355 |
| White, 2009[79] | Chronic kidney disease | <2,500g vs. NBW | OR | 1.73 (1.44 to 2.08) | 8.3 × 10^-9^ | 1.07 (0.92 to 1.25) | 0.078 | 8 | NA | NA |
| Wojcik, 2013[14] | Depression in adulthood | <2,500g vs. ≥2,500g | OR | 1.15 (1.00 to 1.32) | 0.057 | 1.13 (0.94 to 1.37) | 0.096 | 4 | 4.48 | NP |
| Xu, 2009[80] | Breast cancer | Per 1kg increase | OR | 1.08 (1.03 to 1.13) | 2.8 × 10^-3^ | 1.01 (0.93 to 1.10) | 0.043 | 4 | 0.84 | 4.1 × 10^-4^ |
| Yang, 2014[12] | Colorectal cancer | Per 1kg increase | RR | 1.05 (0.93 to 1.19) | 0.461 | 1.01 (0.94 to 1.09) | 0.038 | 1 | 0.27 | 0.149 |
| Yang, 2014[12] | Endometrial cancer | Per 1kg increase | RR | 0.91 (0.81 to 1.03) | 0.128 | 0.89 (0.81 to 0.98) | 0.049 | 2 | 1.68 | 0.758 |
| Yang, 2014[12] | Lung cancer | Per 1kg increase | RR | 1.09 (1.02 to 1.16) | 0.016 | 1.04 (0.96 to 1.13) | 0.042 | 1 | 0.49 | 0.436 |
| Yang, 2014[12] | Melanoma | Per 1kg increase | RR | 1.14 (1.05 to 1.24) | 1.9 × 10^-3^ | 1.13 (1.00 to 1.27) | 0.061 | 2 | 2.25 | NP |
| Yang, 2014[12] | Non-Hodgkin lymphoma | Per 1kg increase | RR | 1.12 (1.01 to 1.24) | 0.033 | 1.15 (1.02 to 1.31) | 0.064 | 1 | 1.31 | NP |
| Yang, 2014[12] | Ovarian cancer | Per 1kg increase | RR | 0.96 (0.88 to 1.04) | 0.295 | 0.92 (0.82 to 1.02) | 0.056 | 0 | 0.96 | NP |
| Zhang, 2013[81] | Diastolic blood pressure | >4,000g vs. NBW | MD | 0.19 (-0.23 to 0.62) | 0.367 | 0.81 (0.40 to 1.21) | 0.207 | 5 | 19.12 | NP |
| Zhang, 2013[81] | Systolic blood pressure | >4,000g vs. NBW | MD | -0.25 (-0.92 to 0.42) | 0.466 | 1.00 (0.48 to 1.52) | 0.265 | 9 | 20.53 | NP |

†Random-effects summary effect size estimated from standardized mean difference transformed to odds ratio

AGA: adequate-for-gestational age, BMC: bone mineral concentration, BMD: bone mineral density, CI: confidence interval, FEV: forced expiratory volume in the first second, HR: hazard ratio, OR: odds ratio, RR: risk ratio, MD: mean difference, RSV: respiratory syncytial virus, SD: standard error, SE: standard error, SGA: small-for-gestational age
